# Supplementary material for: Conformational flexibility is a critical factor in designing broad-spectrum human norovirus protease inhibitors
Source: J Virol. 2025 Jan 28;99(2):e01757-24. doi: 10.1128/jvi.01757-24 (PMC11852804; doi:10.1128/jvi.01757-24)
Supplement: Supplemental material — Figures S1 and S2; Tables S1 to S5. [file jvi.01757-24-s0001.docx]

Supplementary Materials for

**CONFORMATIONAL FLEXIBILITY IS A CRITICAL FACTOR IN DESIGNING BROAD-SPECTRUM HUMAN NOROVIRUS PROTEASE INHIBITORS**

**Son Pham^1^, Boyang Zhao^1,2^, Neetu Neetu^1^, Banumathi Sankaran^3^, Ketki Patil^2^, Sasirekha Ramani^2^, Yongcheng Song^1,4^, Mary K. Estes^2,5^, Timothy Palzkill^1,2^, B.V. Venkataram Prasad^1,2*^**

^1^Verna and Marrs McLean Department of Biochemistry and Molecular Pharmacology, Baylor College of Medicine, Houston, TX, USA

^2^Department of Molecular Virology and Microbiology, Baylor College of Medicine, Houston, TX, USA

^3^Berkeley Center for Structural Biology, Molecular Biophysics, and Integrated Bioimaging, Lawrence Berkeley Laboratory, Berkeley, CA, USA

^4^Dan L Duncan Comprehensive Cancer Center, Baylor College of Medicine, Houston, TX, USA

^5^Department of Medicine, Baylor College of Medicine, Houston, TX, USA

^*^Corresponding author: vprasad@bcm.edu

**This file includes:**

Figures S1 and S2

Tables S1 to S5

## Figure S1


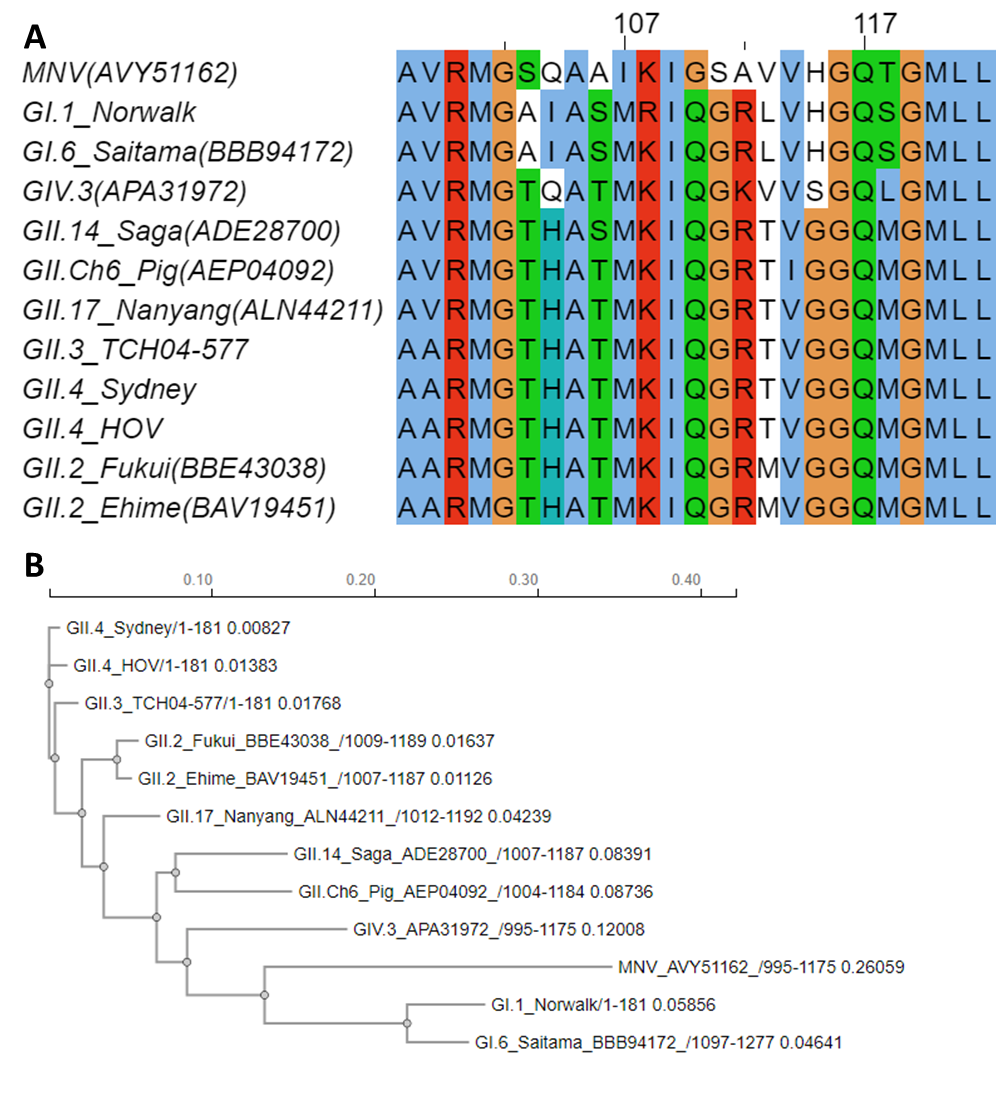


**Figure S1. A)** Protease BII-CII loop sequence alignment of several genogroups and genotypes of norovirus. Loop sequences within each genogroup are largely conserved, especially at positions 112 and 115. **B)** Phylogenetic tree of analyzed protease sequences.

## Figure S2

**Figure S2. Comparison of GII.4 Sydney protease-rupintrivir and GII.4 Sydney-NV-004 structures.** **Left:** GII.4 Sydney is covalently linked to a single molecule of rupintrivir. **Middle:** GII.4 Sydney is linked to one molecule of NV-004 (light green) while binding another NV-004 molecule (pink) in the S4 pocket. **Right:** The CII strand of Sydney-NV-004 structure (gold) is less extended than that of Sydney-rupintrivir structure (indigo), while BII-CII of Sydney-NV-004 is more extended than that of Sydney-rupintrivir structure, likely to match the ligands present in their respective S2 and S4 pockets.

## Table S1: Data collection and refinement statistics for GI.1-Pro-rupintrivir and GII.4-Pro HOV-rupintrivir structures

|  | **GI1_WT_Rupintrivir** | **HOV_WT_Rupintrivir** |
| --- | --- | --- |
| PDB ID | 9D9Y | 9DA7 |
| Wavelength (Å) | 1.00 | 0.9794 |
| Resolution range (Å) | 52.98 - 1.8 (1.83 - 1.8) | 48.99 - 2.5 (2.54 - 2.5) |
| Space group | P 4_3_ 2_1_ 2 | P 3_1_ 2 1 |
| Unit cell (a, b, c, α, β, γ) | 118.464 Å, 118.464 Å, 66.571 Å,  90°, 90°, 90° | 56.569Å, 56.569Å, 95.064Å,  90°, 90°, 120° |
| Total reflections | 384835 (18768) | 79153 (4248) |
| Unique reflections | 44383 (2155) | 6441 (314) |
| Multiplicity | 8.7 (8.3) | 12.3 (13.5) |
| Completeness (%) | 99.95 (99.86) | 99.6 (100) |
| Mean I/sigma(I) | 11.1 (2.6) | 13.7 (2.5) |
| Wilson B-factor (Å^2^) | 23.56 | 43 |
| R-merge | 0.090 (0.834) | 0.096 (0.205) |
| R-meas | 0.096 (0.887) | 0.101 (213) |
| R-pim | 0.032 (0.336) | 0.028 (0.059) |
| CC1/2 | 0.998 (0.761) | 0.997 (0.991) |
| CC* | 1 (0.930) | 0.999 (0.998) |
| Reflections used in refinement | 44363 (2153) | 6439 (313) |
| Reflections used for R-free | 1438 (70) | 326 (18) |
| R-work | 0.1758 (0.2588) | 0.2515 (0.3457) |
| R-free | 0.1993 (0.3071) | 0.2935 (0.5866) |
| CC (work) | 0.962 | 0.934 |
| CC (free) | 0.946 | 0.901 |
| Number of non-hydrogen atoms | 2776 | 1261 |
| macromolecules | 2458 | 1201 |
| ligands | 86 | 43 |
| solvent | 232 | 17 |
| Protein residues | 328 | 159 |
| Nucleic acid bases |  |  |
| RMS (bonds) | 0.008 | 0.005 |
| RMS (angles) | 1 | 1.18 |
| Ramachandran favored (%) | 98.44 | 97.42 |
| Ramachandran allowed (%) | 1.56 | 2.58 |
| Ramachandran outliers (%) | 0 | 0 |
| Rotamer outliers (%) | 0.38 | 0.78 |
| Clashscore | 2.55 | 0.8 |
| Average B-factor (Å^2^) | 29.36 | 46.21 |
| macromolecules | 28.49 | 45.97 |
| ligands | 27.96 | 54.67 |
| solvent | 39.19 | 41.41 |

## Table S2: Data collection and refinement statistics for GII.3-Pro and GII.4-Pro Sydney apo and rupintrivir complex structures

|  | **GII3_WT_Apo** | **GII4_Sydney_Apo** | **GII3_WT**  **_Rupintrivir** | **Sydney_WT**  **_Rupintrivir** |
| --- | --- | --- | --- | --- |
| PDB ID | 9DF5 | 9DEY | 9DAP | 9DAJ |
| Wavelength (Å) | 1.00002 | 1.00002 | 1 | 0.97946 |
| Resolution range (Å) | 63.03 - 2.8 (2.85 - 2.8) | 60.54 - 2.4 (2.44 - 2.4) | 48.66 - 2.71 (2.76 - 2.71) | 48.86 - 2.5 (2.54 - 2.5) |
| Space group | P 4_3_ 2_1_ 2 | C 2 2 2_1_ | P 3_2_ 2 1 | P 3_2_ 2 1 |
| Unit cell (a, b, c, α, β,γ) | 78.525 Å 78.525 Å 105.686 Å,  90° 90° 90° | 112.21Å, 158.9Å, 93.5Å,  90°, 90°, 90° | 97.329Å, 97.329Å, 43.77Å,  90°, 90°, 120° | 97.722 Å, 97.722 Å, 44.62 Å,  90°, 90°, 120° |
| Total reflections | 89910 (4667) | 142215 (7246) | 76702 (4028) | 80229 (3917) |
| Unique reflections | 8595 (415) | 31729 (1586) | 6699 (329) | 3917 (400) |
| Multiplicity | 10.5 (11.2) | 4.5 (4.6) | 11.4 (12.2) | 9.2 (9.8) |
| Completeness (%) | 99.8 (100.0) | 96.1 (98.0) | 99.79 (100.00) | 100.00 (100.00) |
| Mean I/sigma(I) | 18.3 (2.1) | 4.5 (1.9) | 12.62 (2.28) | 19.6 (3.6) |
| Wilson B-factor (Å^2^) | 59.2 | 22.86 | 50.06 | 42.87 |
| R-merge | 0.081 (0.504) | 0.180 (0.468) | 0.1379 (1.046) | 0.090 (0.572) |
| R-meas | 0.085 (0.528) | 0.208 (0.539) | 0.1444 (1.092) | 0.096 (0.604) |
| R-pim | 0.028 (0.157) | 0.102 (0.258) | 0.04211 (0.3096) | 0.04865 (0.3958) |
| CC1/2 | 0.999 (0.915) | 0.798 (0.664) | 0.997 (0.828) | 0.997 (0.761) |
| CC* | 1 (0.977) | 0.942 (0.893) | 0.999 (0.952) | 0.999 (0.930) |
| Reflections used in refinement | 8594 (415) | 30712 (793) | 6685 (329) | 8666 (400) |
| Reflections used for R-free | 430 (21) | 1207 (26) | 318 (14) | 440 (19) |
| R-work | 0.2525 (0.3374) | 0.2261 (0.3233) | 0.1864 (0.3218) | 0.2255 (0.3013) |
| R-free | 0.2973 (0.3936) | 0.2598 (0.4401) | 0.2403 (0.2721) | 0.2746 (0.4039) |
| CC (work) | 0.915 | 0.718 | 0.952 | 0.929 |
| CC (free) | 0.809 | 0.824 | 0.917 | 0.867 |
| Number of non-hydrogen atoms | 2269 | 5346 | 1260 | 1277 |
| macromolecules | 2269 | 5157 | 1217 | 1203 |
| ligands | 0 | 0 | 43 | 51 |
| solvent | 0 | 189 | 0 | 23 |
| Protein residues | 299 | 687 | 161 | 159 |
| Nucleic acid bases |  |  |  |  |
| RMS (bonds) | 0.002 | 0.002 | 0.005 | 0.002 |
| RMS (angles) | 0.57 | 0.56 | 0.77 | 0.63 |
| Ramachandran favored (%) | 94.7 | 98.07 | 98.09 | 98.06 |
| Ramachandran allowed (%) | 5.3 | 1.93 | 1.91 | 1.94 |
| Ramachandran outliers (%) | 0 | 0 | 0 | 0 |
| Rotamer outliers (%) | 0.41 | 0.91 | 0.77 | 0 |
| Clashscore | 2.4 | 2.98 | 2.77 | 1.19 |
| Average B-factor (Å^2^) | 66.74 | 29.88 | 51.27 | 42.98 |
| macromolecules | 66.74 | 30.1 | 51.14 | 42.71 |
| ligands |  |  | 54.81 | 48.84 |
| solvent |  | 23.83 |  | 44.06 |

## Table S3: Data collection and refinement statistics for GII-Pro R112A mutants in complex with rupintrivir

|  | **HOV_R112A**  **_Rupintrivir** | **GII3_R112A**  **_Rupintrivir** | **Sydney_R112A**  **_Rupintrivir** |
| --- | --- | --- | --- |
| PDB ID | 9DA0 | 9DAL | 9D9T |
| Wavelength (Å) | 0.97741 | 0.97741 | 0.97946 |
| Resolution range (Å) | 46.55 - 2.28 (2.32 - 2.28) | 41.16 - 2.6 (2.64 - 2.6) | 42.59 - 2.0 (2.03 - 2.0) |
| Space group | P 3_2_ 2 1 | P 3_2_ 2 1 | P 3_2_ 2 1 |
| Unit cell (a, b, c, α, β,γ) | 93.1 Å, 93.1 Å, 44.66 Å, 90°, 90°, 120° | 95.05Å 95.05Å 46.18Å, 90° 90° 120° | 98.35 Å 98.35 Å 46.28 Å, 90° 90° 120° |
| Total reflections | 106210 (5828) | 136998 (5868) | 327374 (19687) |
| Unique reflections | 9592 (515) | 7621 (378) | 15510 (871) |
| Multiplicity | 11.0 (10.8) | 18.0 (15.5) | 21.1 (22.6) |
| Completeness (%) | 92.1 (100.00) | 100.00 (100.00) | 87.7 (100.00) |
| Mean I/sigma(I) | 23.0 (3.2) | 26.5 (1.8) | 22.3 (3.6) |
| Wilson B-factor (Å^2^) | 41.62 | 71.7 | 41.27 |
| R-merge | 0.079 (0.880) | 0.079 (1.669) | 0.074 (1.077) |
| R-meas | 0.083 (0.922) | 0.081 (1.725) | 0.076 (1.102) |
| R-pim | 0.025 (0.273) | 0.019 (0.433) | 0.016 (0.231) |
| CC1/2 | 0.999 (0.829) | 1 (0.622) | 1 (0.929) |
| CC* | 1 (0.952) | 1 (0.876) | 1 (0.981) |
| Reflections used in refinement | 9589 (515) | 7618 (378) | 15508 (871) |
| Reflections used for R-free | 480 (22) | 768 (36) | 782 (46) |
| R-work | 0.1985 (0.2806) | 0.2232 (0.3290) | 0.2132 (0.2733) |
| R-free | 0.2330 (0.2923) | 0.2475 (0.3412) | 0.2363 (0.2805) |
| CC (work) | 0.938 | 0.948 | 0.845 |
| CC (free) | 0.828 | 0.949 | 0.86 |
| Number of non-hydrogen atoms | 1323 | 1280 | 1304 |
| macromolecules | 1242 | 1237 | 1204 |
| ligands | 43 | 43 | 48 |
| solvent | 38 | 0 | 52 |
| Protein residues | 165 | 165 | 160 |
| Nucleic acid bases |  |  |  |
| RMS (bonds) | 0.002 | 0.008 | 0.004 |
| RMS (angles) | 0.68 | 0.74 | 0.96 |
| Ramachandran favored (%) | 97.52 | 98.14 | 98.08 |
| Ramachandran allowed (%) | 2.48 | 1.86 | 1.92 |
| Ramachandran outliers (%) | 0 | 0 | 0 |
| Rotamer outliers (%) | 0 | 1.52 | 0 |
| Clashscore | 1.93 | 1.17 | 1.19 |
| Average B-factor (Å^2^) | 46.19 | 77.35 | 45.54 |
| macromolecules | 46.31 | 77.27 | 45.43 |
| ligands | 44.73 | 79.69 | 45.73 |
| solvent | 44.24 |  | 47.85 |

## Table S4: BII-CII loop for HuNoV protease structures

| Protein | Average B-factor of residues 106-116 for all chains (Å^2^) | Average B-factor range, by protein monomer (Å^2^) |
| --- | --- | --- |
| GI.1 Apo | 14.7 | N/A |
| GI.1 Rupintrivir | 35.7 | 31.0-40.5 for 2 monomers |
| GII.4 Sydney Apo | 36.0 | 17.8-52.0 for 4 monomers |
| GII.4 Sydney WT Rupintrivir | 46.0 | N/A |
| GII.4 Sydney R112A Rupintrivir | 49.4 | N/A |
| GII.3 Apo | 55.6 | N/A |
| GII.3 WT Rupintrivir | 61.7 | N/A |
| GII.3 R112A Rupintrivir | 84.7 | N/A |
| GII.4 HOV Apo | 68.0 | 51.3-85.1 for 4 monomers |
| GII.4 HOV WT Rupintrivir | 77.8 | N/A |
| GII.4 HOV R112A Rupintrivir | 47.6 | N/A |

## Table S5: Crystallization conditions

| **Crystal** | **Condition** |
| --- | --- |
| GII.3 Apo | 0.1-0.35M KSCN, 15-25% PEG 3,350, 0.1M Bis-Tris Propane pH 7.5 |
| GII.4 Sydney Apo | 1-8% Tacsimate pH 5.0, 4-16% PEG 3,350 |
| GII.3 - Rupintrivir | 0.3 M Sodium Formate, 0.1 M Sodium Citrate Tribasic:HCl, 22.5 % (v/v) PurePEGs Cocktail |
| GII.3 R112A - Rupintrivir | 0.1 M BICINE pH 9.0, 10% (w/v) PEG 20000; 2%(v/v) 1,4-Dioxane |
| GII.4 Sydney - Rupintrivir | 0.3 M Sodium Iodide, 0.1 M Potassium Nitrate:NaOH, 22.5 % (v/v) PurePEGs Cocktail |
| GII.4 HOV - Rupintrivir | 0.1 M BIS-TRIS pH 5.5, 2.0 M Ammonium sulfate |
| GII.4 HOV R112A - Rupintrivir | 0.2 M Lithium acetate dihydrate, 20% w/v Polyethylene glycol 3,350, pH 7.9 |
| GI.1 - Rupintrivir | 0.2 M Lithium sulfate, 0.1M Tris, 1.0 M Sodium/Potassium tartrate, pH 7.0 |
| GII.4 Sydney R112A - Rupintrivir | 0.02 M Magnesium chloride hexahydrate, 0.1M HEPES pH 7.5, 22 % w/v Poly(acrylic acid sodium salt) 5100, 0.2M potassium iodide |
